# Phytoremediation and Nurse Potential of Aloe Plants on Mine Tailings

**DOI:** 10.3390/ijerph20021521

**Published:** 2023-01-13

**Authors:** João Marcelo-Silva, Masego Ramabu, Stefan John Siebert

**Affiliations:** Unit for Environmental Sciences and Management, North-West University, Potchefstroom 2520, South Africa

**Keywords:** restoration, phytoextraction, metals, facilitation

## Abstract

Mine tailings are a source of potentially toxic metals (PTMs) worldwide. Phytoremediation is a low-cost green technology that uses metal-tolerant plants to extract these contaminants and rehabilitate the soil. In mine tailing restoration efforts, it can be beneficial to introduce species that can facilitate the colonization of other plants (i.e., nurse plant syndrome). In this study, the phytoremediation and nursing potential of two species adapted to metalliferous soil, *Aloe burgersfortensis* and *A. castanea*, were evaluated for the first time. An experiment was performed with aloe plants grown in pots containing potting soil, platinum tailings, and gold tailings. Leaves were assessed for bioaccumulation of PTMs. Seeds of Bermuda grass and African daisy, two successional pioneers, were planted with the aloes and had their developmental parameters evaluated after 30 days. Allelopathic effects were also assessed, with seeds of the pioneer plants infused with root extracts of the aloes from the different soil treatments. *A. castanea* demonstrated greater potential for the bioaccumulation of Cd, Co, Mn, Ni, and Zn in the tailings. The presence of aloes benefited germination rates, leaf count, length, and plant biomass of grasses and daisies in the mine tailings, without significant allelopathic effects. Therefore, aloes—especially *A. castanea*—should be employed in the rehabilitation of metal-contaminated soils to extract metals and to aid the establishment of other species to enhance the phytoremediation processes.

## 1. Introduction

The restoration of degraded soils can greatly benefit from the use of nursing plants. These plants (also known as facilitators), are especially important when severe abiotic conditions prevent vegetation establishment [1,2]. They can positively affect the restoration process in several ways—from improving the nutrient cycling in the soil to providing a suitable habitat for seed dispersers [3]. Their application in the rehabilitation of contaminated soils, however, remains to be fully understood [4]. Potentially toxic metals (PTMs), for instance, are abiotic constrains to pioneer plants. These plants could benefit from the presence of species that are not only able to tolerate high metal concentrations (i.e., metallophytes), but also to attenuate the soil conditions, in a facilitative process [5].

South Africa (SA) is currently subjected to intense mining activity, and the mining companies are compelled by law to rehabilitate the resultant degraded environments [6]. Like elsewhere in the world, SA mine tailings are enriched with PTMs from the extraction of minerals and represent a long-term risk for the environment [7]. The rehabilitation of soils contaminated by PTMs has proven to be successful when recognized phytoremediators are employed [8]. These plants can extract metals from the soil and bioaccumulate them in their tissues, with no apparent consequence to their survival [9]. Among candidates, species from the genus *Aloe* have demonstrated potential for phytoextraction of PTMs [10,11,12] and for facilitation of growth of other plants [3]. Some species, such as *A. cryptopoda* Baker, are often transplanted on mine tailings in the Steelpoort region of SA [12].

In this study, we evaluated the potential for phytoremediation and facilitation by *Aloe burgersfortensis* Reynolds and *A. castanea* Schönland grown in mine tailings. There are no reports on such a use for these species to date. They are both edaphic endemic species of SA [13] and commonly used for gardening and medicinal and cosmetic purposes, like most aloes [14]. As for the target species, Bermuda grass (*Cynodon dactylon* (L.) Pers.) and the endemic African daisy (*Dimorphotheca aurantiaca* D.C.) were assessed in terms of early establishment. As pioneers, they are known to rapidly colonize disturbed substrate, promoting soil formation and stabilization [15]. While the impact of PTMs on the development of Bermuda grass is well reported (e.g., [16,17,18]), there are no assessments on the African daisy to date. We consider it essential to investigate the potential of aloes as models for the phytoremediation of contaminated soils and to evaluate the nursing effect that these aloes have on pioneer species.

## 2. Materials and Methods

### 2.1. Soil and Plant Material

Commercially available potting soil mix for succulent plants was used for the control treatment. The treatments containing contaminated soils were composed of tailings from platinum and gold mines, collected in 2018 at the Impala Platinum Mine (25°32.1″ S; 27°10.1″ E) and the Ashanti Gold Mine (26°53.3″ S; 26°52.3″ E), respectively, in the North-West Province, SA. As nursing plants, adult individuals of *A. burgersfortensis* and *A. castanea* were first cultivated from seed in the North-West University Botanical Garden and transplanted to pots containing the control soil and the mine tailings in 2020. They were, therefore, well adapted to the soil conditions after 24 months and suitable for phytoextraction assays. Seeds of Bermuda grass and African daisy, the pioneer species, were acquired from a local gardening store and selected for this study due to their fast growth rates and role as pioneers in natural successional processes.

### 2.2. Pot Experiment

**Figure 1:**
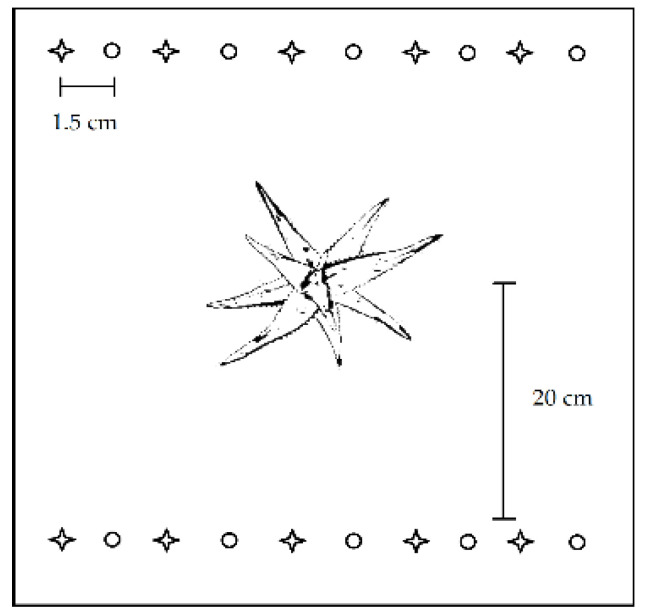
Schematic of seeding placement in a tray used in the pot experiment. Stars = Bermuda grass seeds, circles = African daisy seeds.

Three individuals each of *A. burgersfortensis* and *A. castanea* were planted in individual trays (10 cm × 28 cm × 54 cm) either containing the control soil, platinum, or gold tailings. Trays were placed in a completely random design (∑n_aloes_ = 3 plants × 2 species × 3 treatments = 18 plants) in a greenhouse located at the North-West University, Potchefstroom campus, South Africa. Each pot also received ten seeds each of Bermuda grass and African daisy, planted alternately 20 cm from the base of the aloe plant (∑n_seeds_ = 10 seeds × 2 species × 18 trays = 360 seeds; Figure 1). Germination rate, mortality rate, leaf count, and leaf length were assessed weekly for grasses and daisies. Total biomass (shoots and roots) of the seeded plants was assessed by the end of the experiment. The experiment lasted 30 days, and the trays were exposed to controlled temperature (25 °C), photosynthetically active radiation between 600 and 800 µmol m^−2^ s^−1^, and daily watering of 60 s from a sprinkler system.

### 2.3. Allelopathy Experiment

Extracts of *A. burgersfortensis* and *A. castanea* were obtained by grinding and diluting the roots in distilled water, followed by sieving in a 0.5 mm mesh. Bermuda grass and African daisy seeds were germinated in Petri dishes with filter paper and irrigated daily with either distilled water, 50 or 100 mg mL^−1^ root extract taken from *A. burgersfortensis* or *A. castanea* grown in the control soil, platinum tailings, or gold tailings (∑n_seeds_ = 6 seeds × 3 Petri dishes × 2 target species × 2 aloe species × 2 concentrations × 3 soil treatments + 6 seeds × 3 Petri dishes (distilled water) × 2 target species = 468 seeds). The experiment was also conducted in the greenhouse, with the same time duration and light conditions as the pot experiment.

### 2.4. PTM Analyses

Soils samples (∑n_soil_ = 3 composite samples × 3 treatments = 9 samples) were analyzed with a Thermo Scientific Niton XL3t GOLDD+ handheld X-ray fluorescence spectrometer for the following: As, Cd, Co, Cr, Cu, Mn, Mo, Ni, and Zn. Leaves from each aloe in each treatment were harvested at 5 cm from the apex (∑n_leaves_ = 3 leaves × 3 plants × 2 species × 3 treatments = 54 leaves), oven-dried at 40 °C, and assessed for the same PTMs as in the soils with an ICP-MS (Agilent 7500 series, Santa Clara, CA, USA) calibrated with a multielement solution (PerkenElmer Pure). The sample preparation method consisted of grinding the leaves and weighing approximately 50 mg of each, followed by acid digestion with HNO_3_ (9 mL, 65%) and HCl (3 mL, 32%), and finally 15 min in a 200 °C microwave (Milestone, Ethos UP, Maxi 44, Bumby, Denmark).

### 2.5. Data Analysis

To test the hypotheses of variation in PTM concentration in aloe leaves and variation of developmental parameters in pioneer species, data were submitted to Permutational Multivariate Analyses of Variances (Permanova; Euclidian distance; 5% significance). This nonparametric method, although somewhat limited in detecting significant differences, allowed to counter possible effects of the small sample sizes and non-normality of data distribution (Shapiro–Wilks, 5% significance; [19]). PTM concentration in the leaves of aloes grown in the different soil treatments had their values evaluated individually, as variables, in 2-way Permanova analyses using as factors “treatments” (3 levels: control, platinum tailings, and gold tailings) and “species” (2 levels: *A. burgersfortensis* and *A. castanea*). The early development of Bermuda grass and African daisy was evaluated individually in terms of germination and mortality rates, leaf count, leaf length, and plant biomass. The design was the same as for the metals in aloes, where the levels in the “species” factor were now the nursing plants. Pairwise comparisons were applied between and within treatments in both PTM and developmental designs, with Monte Carlo tests when permutations were fewer than 100 [19]. Regression curves were calculated in the search of trends among treatments when the presence of aloes was not considered. The germination rates from the allelopathy experiment were tested in a 1-way Permanova design, for each of the target species, using the root extracts of the aloe species from different soils as the factor “treatments” (13 levels: control soil/platinum/gold tailings × *A. burgersfortensis* 50 and 100 mg mL^−1^ and *A. castanea* 50 and 100 mg mL^−1^ and distilled water). The bioaccumulation factor (BAF) was calculated as a measure of the average of PTMs in the leaves of the aloes divided by the average of the PTMs in the soils from each treatment. Ratios above 1 were considered as bioconcentration (i.e., phytoextraction) of PTMs [20].

## 3. Results

**Figure 2:**
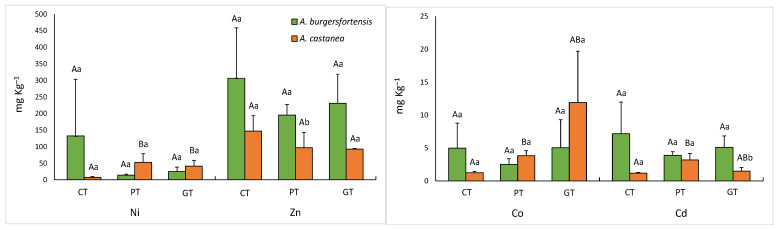
Mean values (± std) of significantly different PTMs in leaves of *A. burgersfortensis* and *A. castanea*. Different upper-case letters: significant difference (*p* < 5%) for a PTM in the same aloe species in different soil treatments. CT = control, PT = platinum tailings, and GT = gold tailings. Different lower-case letters: significant difference for a PTM between aloe species in the same soil treatment.

Soils had different degrees of contamination (Appendix A) and influenced the concentration and bioaccumulation of PTMs in leaves of the aloes (Figure 2; Table 1). The contaminated soils also contributed to a decreasing trend in most of the developmental parameters of Bermuda grass and African daisy over time.

### 3.1. PTM Concentration and Metal Transfer Factors in Aloes

Ni, Zn, Co, and Cd differed significantly in the leaves of aloes from the different soil treatments (Figure 2). By observing the metal uptake in each species separately, it was noted that *A. castanea* had higher levels of Ni in both platinum and gold tailings, when compared to the control soil (t = 0.10; *p* = 0.039 and t = 0.10; *p* = 0.028, respectively). In addition, Co and Cd were also significantly higher in *A. castanea* leaves from the platinum tailings compared to the control (t = 0.10; *p* = 0.004 and t = 0.10; *p* = 0.021, respectively). Comparing the two species with one another per soil treatment revealed that the Zn concentrations were significantly higher in *A. burgersfortensis* than in *A. castanea* only in the platinum tailings (t = 0.10; *p* = 0.039), whereas the same was found for Cd only in the gold tailings (t = 0.10; *p* = 0.025). *A. burgersfortensis* bioaccumulated Cd and Zn in all soil types and Mn only in the control soil (Table 1), whereas *A. castanea* bioaccumulated Cd, Mn, Ni, and Zn only in the contaminated soils.

### 3.2. Early Development and Allelopathic Effects on Bermuda Grass and African Daisy

**Figure 3:**
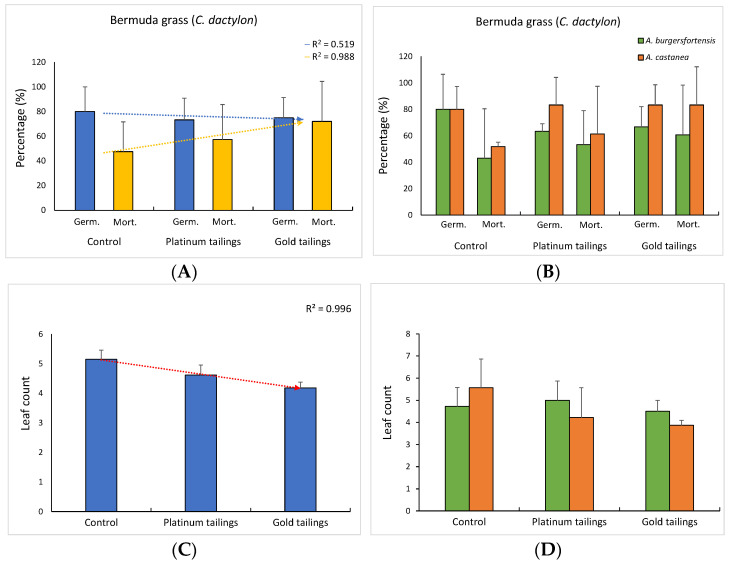

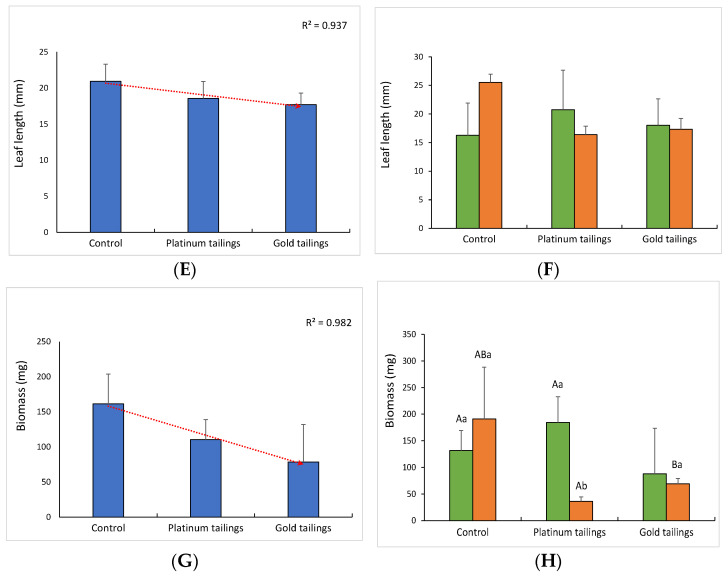
Mean values (± sd) and regression curves for developmental parameters of Bermuda grass considering only the soil treatments (**A**,**C**,**E**,**G**) and the soil treatments in the presence of aloes (**B**,**D**,**F**,**H**). Different upper-case letters: significant differences (*p* < 5%) among individuals in different soil treatments in the presence of the same aloe species. Different lower-case letters: significant differences among individuals grown with different aloe species, in the same soil treatment.

**Figure 4:**
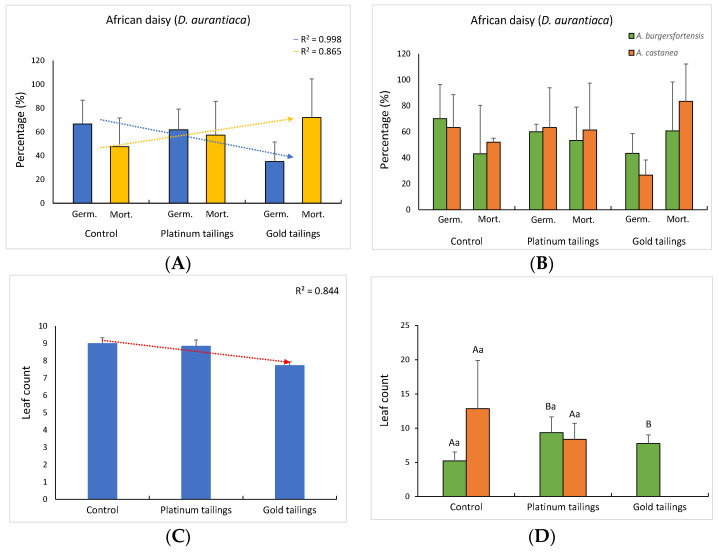

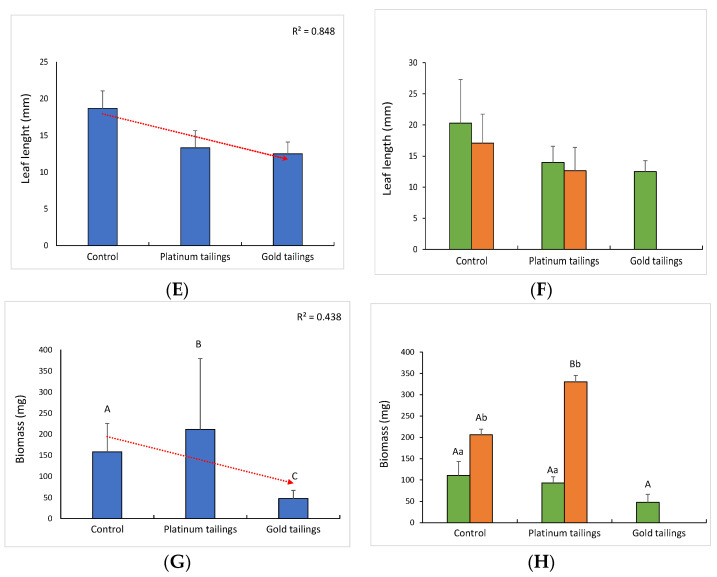
Mean values (± sd) and regression curves for developmental parameters of African daisy considering only the soil treatments (**A**,**C**,**E**,**G**) and the soil treatments in the presence of aloes (**B**,**D**,**F**,**H**). Different upper-case letters: significant differences (*p* < 5%) among individuals in different soil treatments in the presence of the same aloe species. Different lower-case letters: significant differences among individuals grown with different aloe species, in the same soil treatment.

The biomass of African daisy was the only parameter affected by the different soil treatments, despite the presence of aloes, with each soil reflecting a distinct effect on the plants (control × platinum: t = 4.10; *p* = 0.012; control × gold: t = 3.34; *p* = 0.029; platinum × gold: t = 2.97; *p* = 0.043). All other parameters only presented nonsignificant trends for reduction in both Bermuda grass and African daisy parameters, while the opposite was observed for the mortality rates (Figure 3A and Figure 4A). When the presence of *A. burgersfortensis* was considered, the trend for a reduction in parameters for Bermuda grass in contaminated soil was not observed, except for germination. Nevertheless, the biomass was significantly reduced only in the gold tailings, for grasses with *A. castanea* (t = 4.34; *p* = 0.013). For the daisies, the trend for a decrease in the leaf count was reversed when planted with *A. burgersfortensis*. In addition, the biomass increased significantly from the control soil to the platinum tailings in the presence of *A. castanea* (t = 10.75; *p* = 0.001; Figure 4H).

**Figure 5:**
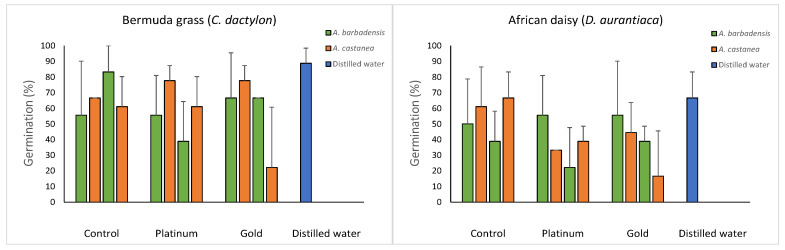
Germination rates (mean ± sd) of seeds from the target species treated with different concentrations of roots extracts of aloes from different soil treatments.

There were no significant differences in seed germination for grasses or daisies with the application of different root extracts of aloes from different soils (Figure 5). However, concentrations of 100 mg mL^−1^ of *A. burgersfortensis* grown in platinum tailings and *A. castanea* grown in gold tailings resulted in germination averages below 40% for both target species.

## 4. Discussion

Studies on the phytoextraction of PTMs by aloes have demonstrated their potential for phytoremediation [12,21,22]. The uptake and the bioaccumulation in leaves, however, depend on the aloe species, the type and concentration of PTMs available in the soil, and the geographical conditions [10,23]. Although the evidence for phytoextraction of PTMs by aloes has been increasing, their use in phytoremediation or restoration of degraded areas is still far from being a thematic research domain for the genus [24]. This is the first study to evaluate the concentrations of PTMs and bioaccumulation in *A. burgersfortensis* and *A. castanea* (both metallophytes from harsh ultramafic soil), and it is reasonable to assume that they are influenced by the same factors mentioned above. Between these species, *A. castanea* would be a better option for phytoremediation given its bioaccumulation of Cd, Mn, Ni, and Zn in contaminated soils. It is important to note that soil modifiers (e.g., calcium carbonate and mushroom residues) can improve the phytoremediation potential of plants and should also be considered [25].

Aloes are also drought -tolerant and are able to create beneficial microhabitats [26,27] and act as nurse plants [3] for the establishment of other species, which are not metal-tolerant. The contaminated soils of this study negatively influenced the development of Bermuda grass, and this is in accordance with previous reports (e.g., [18,28,29]). Nevertheless, the fact that the biomass was the only parameter to significantly decrease under contamination points to the tolerance of this grass to PTMs in the soil and reinforces its recommendation for rehabilitation and phytostabilization of contaminated areas [30,31,32]. The development of the African daisy was also compromised in the contaminated soils, especially in the gold tailings, where all seedlings died before full development of the first leaves, in the treatment with *A. castanea*. The increase in biomass of the daisy in the platinum tailings with *A. castanea*, however, suggests that the tolerance might be dependent on both the soil features and the nursing plant. There are no records of development evaluation of the African daisy grown in contaminated soils. However, the closely related Cape marguerite (*D. eckloni* DC.), also an endemic South African species, showed potential for phytoextraction and an increase in growth parameters under Cd contamination [33]. Many members of the Asteraceae are known to be suitable for the phytoremediation of contaminated areas [34].

The facilitation process demonstrated here was dependent on the levels of soil contamination, the type of nursing species (i.e., *A. burgersfortensis* or *A. castanea*), and the target pioneer species (i.e., *C. dactylon* and *D. aurantiaca*). These and other environmental features may define if the interaction between putative nurse and target plants will be facilitative or competitive [1]. Our trials provided evidence that there was a negative influence of contaminated soils on Bermuda grass and African daisy growth and development and that such an influence was attenuated by the presence of aloes. In addition, the absence of allelopathic effects from the root extracts of the aloes on the seed germination of both the grass and daisy showed that aloe roots do not exudate harmful components into the substrate. In fact, the few reports of allelopathy on germination promoted by aloes are mainly based on leaf extracts and give contradictory results [35,36,37], with concentrations way lower than those used in this study.

## 5. Conclusions

*Aloe burgersfortensis* and *A. castanea* showed potential for the phytoextraction and bioaccumulation of PTMs, especially in contaminated soils from platinum and gold tailings. They also attenuated the negative impacts of these contaminated soils on the early development of Bermuda grass and African daisy in pot experiments. The root extract of aloes did not affect the germination of these target species. This is the first time that this facilitation process has been demonstrated and must, therefore, be explored further in field experiments, especially when aiming for the rehabilitation and restoration of contaminated soils. These findings have a major application for the remediation of mine tailings of semiarid areas in Africa. Aloes are native, preadapted species that can be successfully included into rehabilitation processes to not only extract PTMs from the soil, but also facilitate the establishment and survival of critical pioneer species.

## Figures and Tables

**Table 1:** Bioaccumulation factors for the aloe plants in the different soil treatments. Bold values: BAF > 1.

	As	Zn	Cu	Ni	Co	Fe	Mn	Cr	Cd
*A. burgersfortensis*									
Control	0.1	**1.3**	0.1	0.4	0.0	0.0	**2.0**	0.0	**2.2**
Platinum tailings	0.0	**1.2**	0.1	0.1	0.0	0.0	0.7	0.0	>1 *
Gold tailings	0.0	**1.7**	0.1	0.1	0.0	0.0	0.9	0.0	**1.5**
*A. castanea*									
Control	0.1	0.6	0.1	0.1	0.0	0.0	0.8	0.0	0.4
Platinum tailings	0.1	**3.0**	0.6	**1.3**	0.0	0.0	**2.6**	0.0	>1 *
Gold tailings	0.1	**3.5**	0.6	0.9	0.2	0.0	**1.8**	0.0	0.4

* Concentration below limit of detection in the soil.

## Data Availability

The dataset is available from the authors by request.

## Appendix A

**Table A1:** PTMs concentrations (Mean ± Std) in soil, *A. Burgersfortensis* and *A. Castanea* leaves under the study treatments. Values in bold are above permissible limits [38].

	Control	Platinum Tailings	Gold Tailings
*Soil*			
As	2.24 ± 4.57	**17.10 ± 4.87**	**16.98 ± 4.15**
Cd	3.26 ± 8.07	<LOD	3.41 ± 9.93
Co	115.64 ± 103.9	**413 ± 111.73**	**371.60 ± 119.39**
Cr	3012 ± 36.23	11,756 ± 72.99	14,470.17 ± 87.24
Cu	**111.18 ± 14.14**	**151.46 ± 16.99**	**137.37 ± 17.19**
Mn	**891.69 ± 73.80**	**1000.54 ± 117.65**	**1083.34 ± 134.11**
Mo	1.69 ± 3.23	6.32 ± 3.57	<LOD
Ni	56.05 ± 17.59	**204.87 ± 22.91**	**230.53 ± 41.15**
Zn	235.01 ± 12.30	161.42 ± 11.60	134.34 ± 11.26
pH	7.24	6.70	7.21
*A. burgersfortensis*			
As	0.25 ± 0.02	0.34 ± 0.06	0.25 ± 0.05
Cd	7.17 ± 4.81	3.88 ± 0.56	5.10 ± 1.76
Co	4.16 ± 4.95	2.51 ± 0.85	5.04 ± 4.28
Cr	9.47 ± 4.89	12.76 ± 14.51	3.74 ± 0.98
Cu	15.32 ± 2.87	18.17 ± 7.61	16.19 ± 1.77
Mn	1399.30 ± 604.86	681.83 ± 235.03	650.65 ± 5.04
Mo	0.06 ± 0.00	0.09 ± 0.13	0.05 ± 0.05
Ni	33.50 ± 15.45	14.51 ± 2.62	25.33 ± 12.97
*A. castanea*			
As	0.26 ± 0.06	0.19 ± 0.02	0.27 ± 0.06
Cd	1.18 ± 0.13	3.21 ± 0.96	1.49 ± 0.57
Co	1.24 ± 0.21	3.83 ± 0.76	11.93 ± 7.78
Cr	5.42 ± 2.41	3.94 ± 0.45	3.24 ± 0.42
Cu	13.93 ± 2.70	18.94 ± 9.24	15.24 ± 3.34
Mn	714.37 ± 400.89	519.73 ± 272.21	393.60 ± 252.94
Mo	0.08 ± 0.09	0.04 ± 0.07	0.05 ± 0.06
Ni	7.91 ± 2.40	52.94 ± 25.88	41.54 ± 17.26
Zn	147.23 ± 46.51	96.81 ± 46.79	92.71 ± 2.60

## References

[1] Padilla F.M., Pugnaire F.I. (2006). The Role of Nurse Plants in the Restoration of Degraded Environments. Front. Ecol. Environ..

[2] Pugnaire F.I., Luque M.T. (2001). Changes in Plant Interactions along a Gradient of Environmental Stress. Oikos.

[3] King E.G., Stanton M.L. (2008). Facilitative Effects of Aloe Shrubs on Grass Establishment, Growth, and Reproduction in Degraded Kenyan Rangelands: Implications for Restoration. Restor. Ecol..

[4] Krumins J.A., Goodey N.M., Gallagher F. (2015). Plant–Soil Interactions in Metal Contaminated Soils. Soil Biol. Biochem..

[5] Wang J., Ge Y., Chen T., Bai Y., Qian B.Y., Zhang C.B. (2014). Facilitation Drives the Positive Effects of Plant Richness on Trace Metal Removal in a Biodiversity Experiment. PLoS ONE.

[6] Republic of South Africa National Environmental Management Amendment Act 62 of 2008. https://www.gov.za/documents/national-environmental-management-amendment-act-2.

[7] Adhikari S., Marcelo-Silva J., Beukes J.P., van Zyl P.G., Coetsee Y., Boneschans R.B., Siebert S.J. (2022). Contamination of Useful Plant Leaves with Chromium and Other Potentially Toxic Elements and Associated Health Risks in a Polluted Mining-Smelting Region of South Africa. Environ. Adv..

[8] Schwitzguébel J.-P., Van Der Lelie D., Baker A., Glass D.J., Vangronsveld J. (2002). Phytoremediation: European and American Trends. J. Soil Sediments.

[9] Van der Ent A., Baker A.J.M., Reeves R.D., Pollard A.J., Schat H. (2013). Hyperaccumulators of Metal and Metalloid Trace Elements: Facts and Fiction. Plant Soil.

[10] Rai S., Sharma D.K., Arora S.S., Sharma M., Chopra A.K. (2011). Concentration of the Heavy Metals in *Aloe vera* L. (*Aloe barbadensis* Miller) Leaves Collected from Geographical Locaion of India. Ann. Biol. Res..

[11] Tanee F.B., Amadi N. (2016). Screening of *Polyalthia longifolia* and *Aloe vera* for Their Phytoextractability of Heavy Metals in Tropical Soil of the Niger Delta. J. Appl. Sci. Environ. Manag..

[12] Dobbins D.C., Marcelo-Silva J., Siebert S.J. (2021). Screening the Phytoextractability of Trace Metals by *Aloe cryptopoda* Baker and *Aloe vera* (L.) Burm.f. Cultivated on Mine Tailings. S. Afr. J. Bot..

[13] Siebert S.J., Van Wyk A.E., Bredenkamp G.J. (2001). Endemism in the Flora of Ultramafic Areas of Sekhukhuneland, South Africa Endemism in the Flora of Ultramafic Areas of Sekhukhuneland. S. Afr. J. Sci..

[14] Smith G. (2011). Sasol First Field Guide to Aloes of Southern Africa.

[15] Arocena J.M., Van Mourik J.M., Schilder M.L.M., Faz Cano A. (2010). Initial Soil Development Under Pioneer Plant Species in Metal Mine Waste Deposits. Restor. Ecol..

[16] Xie Y., Hu L., Du Z., Sun X., Amombo E., Fan J., Fu J. (2014). Effects of Cadmium Exposure on Growth and Metabolic Profile of Bermudagrass [*Cynodon dactylon* (L.) Pers.]. PLoS ONE.

[17] Delgado-Caballero M.d.R., Alarcón-Herrera M.T., Valles-Aragón M.C., Melgoza-Castillo A., Ojeda-Barrios D.L., Leyva-Chávez A. (2017). Germination of *Bouteloua dactyloides* and *Cynodon dactylon* in a Multi-Polluted Soil. Sustainability.

[18] Song X., Li C., Chen W. (2022). Phytoremediation Potential of Bermuda Grass (*Cynodon dactylon* (L.) Pers.) in Soils Co-Contaminated with Polycyclic Aromatic Hydrocarbons and Cadmium. Ecotoxicol. Environ. Saf..

[19] Anderson M., Gorley R.N., Clarke K.R. (2008). PERMANOVA+ for PRIMER: Guide to Software and Statistical Methods.

[20] Cluis C. (2004). Junk-Greedy Greens: Phytoremediation as a New Option for Soil Decontamination. BioTeach J..

[21] Shokri F., Ziarati P., Mousavi Z. (2016). Removal of Selected Heavy Metals from Pharmaceutical Effluent by *Aloe vera* L. Biomed. Pharmacol. J..

[22] Elhag M., Al-Ghamdi A.A.M., Galal H.K., Dahlan A. (2018). Evaluation of *Aloe vera* L. as Phytoremediator of Heavy Metals Contaminated Soils in Arid Environments. Appl. Ecol. Environ. Res..

[23] Ong G.H., Cheng W.H., Wong L.S. (2018). Accumulation of Arsenic and Antimony in *Aloe barbadensis*: A Transplantation Study. Remediation.

[24] Adetunji T.L., Olisah C., Adegbaju O.D., Olawale F., Adetunji A.E., Siebert F., Siebert S. (2022). The Genus Aloe: A Bibliometric Analysis of Global Research Outputs (2001–2020) and Summary of Recent Research Reports on Its Biological Activities. S. Afr. J. Bot..

[25] Su R., Ou Q., Wang H., Luo Y., Dai X., Wang Y., Chen Y., Shi L. (2022). Comparison of Phytoremediation Potential of *Nerium indicum* with Inorganic Modifier Calcium Carbonate and Organic Modifier Mushroom Residue to Lead–Zinc Tailings. Int. J. Environ. Res. Public Health.

[26] Klopper R.R., Smith G.F. Aloe Genus. http://pza.sanbi.org/aloe-genus.

[27] Tariq M.R., Shaheen F., Mustafa S., Sajid A.L.I., Fatima A., Shafiq M., Safdar W., Sheas M.N., Hameed A., Nasir M.A. (2022). Phosphate Solubilizing Microorganisms Isolated from Medicinal Plants Improve Growth of Mint. PeerJ.

[28] Xie Y., Luo H., Hu L., Sun X., Lou Y., Fu J. (2014). Classification of Genetic Variation for Cadmium Tolerance in Bermudagrass [*Cynodon dactylon* (L.) Pers.] Using Physiological Traits and Molecular Markers. Ecotoxicology.

[29] Xiong Z., Yang J., Zhang K. (2021). Effects of Lead Pollution on Germination and Seedling Growth of Turfgrass, *Cynodon dactylon*. Pakistan J. Bot..

[30] Shu W.S., Ye Z.H., Lan C.Y., Zhang Z.Q., Wong M.H. (2002). Lead, Zinc and Copper Accumulation and Tolerance in Populations of *Paspalum distichum* and *Cynodon dactylon*. Environ. Pollut..

[31] Archer M.J.G., Caldwell R.A. (2004). Response of Six Australian Plant Species to Heavy Metal Contamination at an Abandoned Mine Site. Water. Air. Soil Pollut..

[32] Shuaibu L., Abdullahi U., Yaradua A.I., Bungudu J.I. (2021). Phytoremediation Potentials of *Cynodon dactylon* on Heavy Metal Contaminated Soils from Challawa Industrial Estate, Kano-Nigeria. Asian J. Appl. Chem. Res..

[33] El-Sayed S., Abdel-Aziz N., Mazhar A. (2022). Antioxidant Isoenzymes, Chemical Constituents and Growth Parameters of Cad-mium-stressed *Dimorphotheca ecklonis* Plant and Affected by Humic Acid. Egypt. J. Chem..

[34] Nikolić M., Stevović S. (2015). Family Asteraceae as a Sustainable Planning Tool in Phytoremediation and Its Relevance in Urban Areas. Urban For. Urban Green..

[35] Ravlić M., Baličević R., Visković M., Smolčić I. (2017). Response of Weed Species on Allelopathic Potential of *Aloe vera* (L.) Burm.F. Herbol. Int. J. Weed Res. Control.

[36] Baličević R., Ravlić M., Lucić K., Tatarević M., Lucić P., Marković M. (2018). Allelopathic Effect of *Aloe vera* (L.) Burm.F. on Seed Germination and Seedlings Growth of Cereals, Industrial Crops and Vegetables. Poljoprivreda.

[37] Singh M., Shankar H., Singh K. (2019). The Allelopathy Effect of Medicinal Crop (*Aloe vera* (L.) Burm.F.) On Their Associated Weeds in Grid Zone of Madhya Pradesh. J. Rural Agric. Res..

[38] Department of Environmental Affairs (2012). National Environmental Management: Waste Act, 2008 (Act no. 59 of 2008), Draft National Norms and Standards for the Remediation of Contaminated Land and Soil Quality. Government Gazette. https://cer.org.za/wp-content/uploads/2010/03/national-environmental-management-waste-act-59-2008-national-norms-and-standards-for-the-remediation-of-contaminated-land-and-soil-quality_20140502-GGN-37603-00331.pdf.

